# Psoriasis: A Primer for General Physicians

**DOI:** 10.7759/cureus.38037

**Published:** 2023-04-24

**Authors:** Shalini Dasari, Ankita Choudhary, Bhushan Madke

**Affiliations:** 1 Dermatology, Jawaharlal Nehru Medical College, Datta Meghe Institute of Higher Education and Research, Wardha, IND; 2 Dermatology, B.J. Medical College, Ahmedabad, IND; 3 Dermatology, Venereology and Leprosy, Jawaharlal Nehru Medical College, Datta Meghe Institute of Higher Education and Research, Wardha, IND

**Keywords:** pathogenesis of psoriasis, sphingosine 1-phosphate receptor 1, inflammatory skin disease, il17, il23, dendritic cells jak inhibitors, psoriasis

## Abstract

Psoriasis is a multisystem, polygenic, inflammatory condition that typically causes changes in the skin. Although there is a significant genetic component, environmental factors like infections can have a significant impact on triggering the disease. A major part of the pathogenesis of psoriasis is played by the Interleukin (IL) IL23/IL17 axis along with the immune-related cells mainly macrophages and dendritic cells (DCs). Additionally, the role of various cytokines along with the toll-like receptors has also been pointed out in immunopathogenesis. These have been supported by the efficacy of biological therapies including TNF alpha inhibitors and inhibitors of IL17 and IL23. We have summarized the topical as well as systemic therapies for psoriasis including biologics. The article throws light on a few emerging therapeutic options like modulators of sphingosine 1-phosphate receptor 1 and Rho-associated kinase 2 inhibitors.

## Introduction and background

Psoriasis is a multisystem, polygenic inflammatory condition predominantly affecting the skin. It is categorized under the papulosquamous group of cutaneous disorders which can present in any age group. The onset of the disease is mainly affected by environmental factors and genetic susceptibility. The prevalence of psoriasis worldwide is found to be roughly 2-3% with a slightly higher male preponderance [1]. The highest peak age of onset of psoriasis in adults varies between the third and fourth decades of life [2,3].

The various systemic associations of psoriasis include obesity, hypertension, metabolic syndrome, inflammatory bowel disease, cardiovascular disease, and chronic renal disease. Patients with psoriasis may also experience psychosocial problems both psychotic and neurotic. Highly demarcated erythematous plaque along with silvery white micaceous scale is a prototype cutaneous lesion of psoriasis, however, sterile pustules can also be seen in some patients. Commonly main sites of involvement are the scalp, elbow, knees, nails, and trunk. Extensor involvement is more common though flexors also get involved in extensive psoriasis. Histologically psoriasis is characterized by hyperkeratosis, parakeratosis, Munro's microabscess, regular elongation of rete ridges, dilated blood vessels, an inflammatory infiltrate of leucocytes in both the epidermis as well as dermis and conspicuous blood vessels of the dermis The various clinical variants include pustular psoriasis, plaque psoriasis, guttate psoriasis, and erythrodermic psoriasis. The various site-specific variants include scalp psoriasis, palmoplantar psoriasis, flexural/inverse psoriasis, nail psoriasis, mucosal psoriasis, sebopsoriasis, genital psoriasis, and psoriatic arthritis. The current review intends to throw light on the pathogenesis of psoriasis, the new and latest concepts in its pathophysiology, and summarizes the various treatment modalities available both traditional as well as upcoming.

## Review

Search methodology

**Figure 1:**
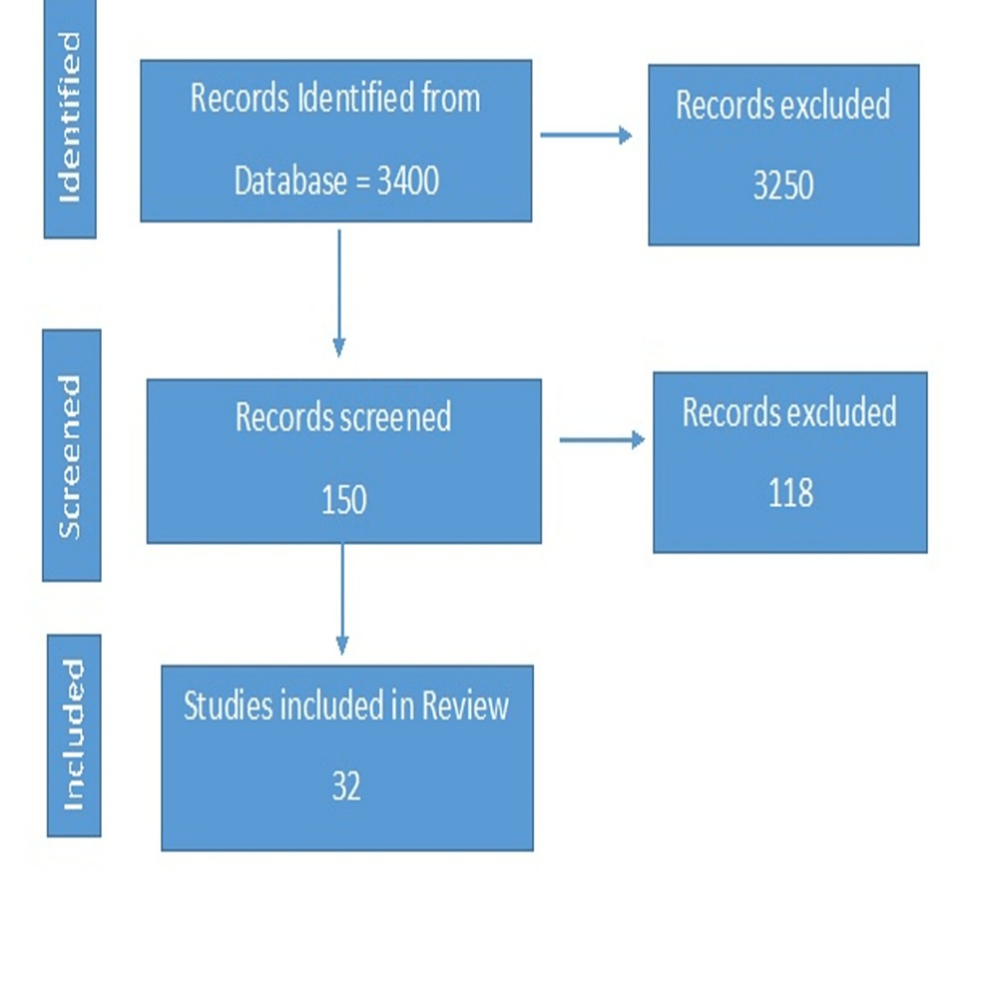
PRISMA flowchart for article search PRISMA: Preferred Reporting Items for Systematic Reviews and Meta-Analyses

We undertook a systematic search through PubMed and CENTRAL in November 2020 using keywords such as "psoriasis", "interleukins", "psoriasis" [Title/Abstract] AND "interleukin" [Title/Abstract]. We additionally searched for key references from bibliographies of the relevant studies. One reviewer independently monitored the retrieved studies against the inclusion criteria, in the beginning, based on the title and abstract and then on full texts. Another reviewer also reviewed approximately 20% of these studies to validate the inclusion of studies. Figure 1 shows the PRISMA flowchart of the search.

Pathophysiology of psoriasis

**Figure 2:**
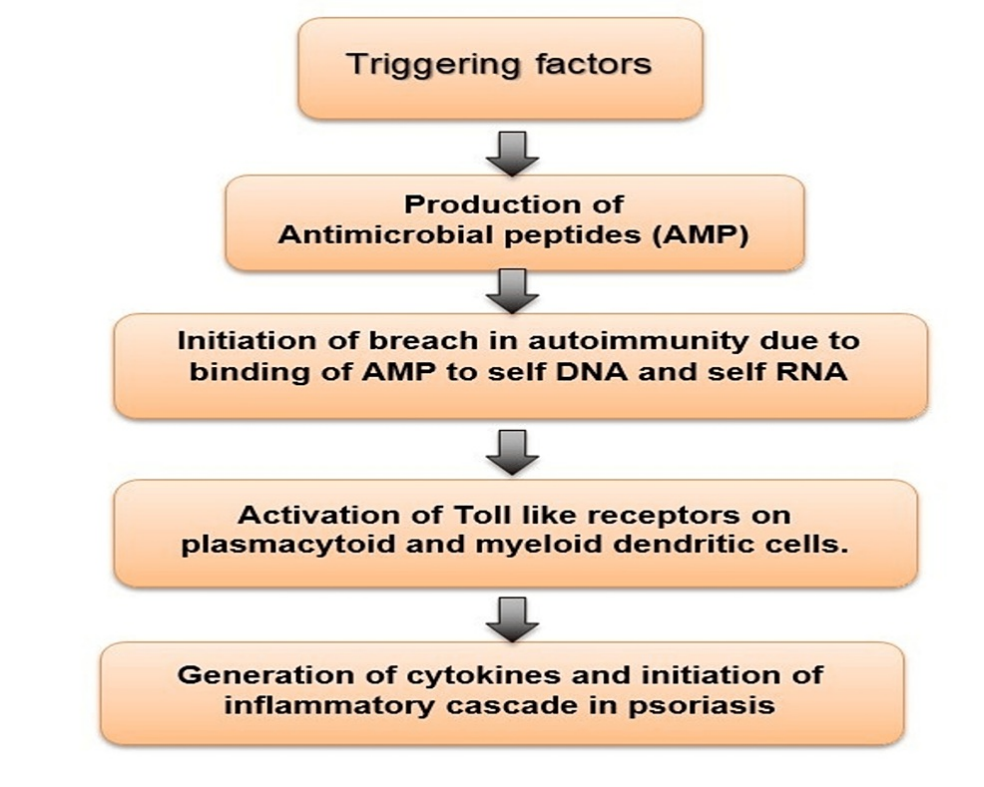
Sequence of events after exposure to a triggering factor

Psoriasis is the complex interplay among the T cell irregularities and the interactions between keratinocytes and the complex cytokine network. The dysfunctional CD4 helper T cells (Th1, Th17, Th 22, and Treg cells) are predominantly involved in the immunopathogenesis of psoriasis [4]. Role of genetic factors: The numerous pieces of evidence implicate that there is a substantive role of genetic factors with respect to susceptibility and disease expression [5]. Genome-wide association studies have revealed nine genomic loci PSORS1-PSORS9 (psoriasis susceptibility) [6]. The most important gene associated is PSOR1, located on the HLA class1 region on chromosome 6p, which accounts for 50% of the risk. Figure 2 shows the sequence of events leading to the initiation of an inflammatory cascade following an inciting factor.

Type 1 psoriasis is characterized by a positive family history, early onset of the disease, and expression of HLA-CW06 in contrast to type 2 psoriasis, which has an absence of family history, a later age of onset, and lack of expression of HLA-CW06.

Triggering factors

The expression of the disease occurs in genetically predisposed individuals in the presence of inciting agents mentioned below: Physical trauma/Injury to the skin leads to the elicitation of psoriatic lesions and hence the Koebner phenomena. Trauma induces the release of neuropeptides from the cutaneous sensory nerve terminals. A few common drugs which can cause exacerbation of psoriasis include lithium, beta-blockers, antimalarial, non-steroidal anti-inflammatory drugs (NSAIDs), and terbinafine [7]. Imiquimod via activation of toll-like receptors 7 can lead to the production of interferons and can trigger psoriasis. Infection with streptococcus can trigger guttate psoriasis via molecular mimicry due to the close resemblance of streptococcal M protein with type 1 keratin expressed on keratinocytes [8]. HIV can alter the course and severity of psoriasis and cause exacerbation via various mechanisms, such as the increased activity of CD8 cells and also low CD4 count with loss of T regulatory cells. Stress and the release of substance P and nerve growth factors from the unmyelinated terminals of cutaneous sensory nerve fibers can lead to neurogenic inflammation in those who are genetically predisposed [9]. Obesity and metabolic syndrome has a close association with psoriasis. Smoking strongly affects palmoplantar pustulosis [10]. Alcoholism is associated with more severe psoriasis and also contributes to the increased probability of cardiovascular disease. Endocrine factors like hypocalcemia serve as the main factor for triggering generalized pustular psoriasis. Pregnancy can alter the disease activity and can lead to impetigo herpetiformis, which is the pustular psoriasis of pregnancy.

Immunopathogenesis

Role of Cytokines and Chemokines

Psoriasis is a disease with the main involvement of the T helper cell subset and their secreted cytokines. There is an overproduction of Th1 cytokines (IFN-Gamma, Il-2, TNF-alpha) while anti-inflammatory cytokines, e.g. IL-10, are decreased. IL-23, produced by plasmacytoid dendritic cells (DCs), initiates Th17 cells to produce cytokines, mainly IL17 and IL22. All these cytokines lead to keratinocyte proliferation and dermal inflammation. The circulating levels of IL22 relate to the severity of the disease. A special subset of IL22-producing T helper cells (Th22 cells) also contributes to psoriasis pathogenesis [11]. IFN-Y released by NK cells and activated T cells in the epidermis activate STAT transcription factor, and this IFN-Y-activated pathway is the main feature of psoriasis. This pathway is responsible for vasodilation by induction of nitric oxide synthase and collection of T cells.

The innate immune cytokines (IL-6, IL-1 TNF-alpha) are upregulated in psoriatic skin. The regulatory T cells, which normally prohibit the formation of an inflammatory loop, are absent in psoriasis. Chemokines are important mediators in the trafficking of leucocytes [12]. Examples are CXCL8 (mediates infiltration by neutrophils), CCL17, CCL20, CCL2, CXCL9-11: Attracts T cells to psoriatic plaques, Chimerin: Contributes to early recruitment of plasmacytoid DCs into psoriatic lesions.

Role of Tumor Necrosis Factor (TNF)

TNF, classically considered a Th1 cytokine, is released by a lot many cells in the inflammatory cascade but also works synergistically with many other cytokine effectors [13].

CD11C dermal DCs and CD163 macrophages are the two most important cells responsible for producing TNF-alpha in the psoriatic plaques.

The various roles played by TNF include the upregulation of intracellular adhesive molecules on the endothelial cell surface and is chemotactic for neutrophils, potentiation of Th1 response in inflammatory reactions, activation of DCs, production of IL-23, keratinocyte proliferation in synergy with IL-22, stimulation of IL-22R, activation of transcription factor-NFKB.

IL23/Th17 Pathway

IL23 is a heterodimeric cytokine composed of p40 and p19 subunits and binds to a receptor complex encoded by IL23R and IL12RB1. IL17 and IL22 reduce keratinocyte expression of anti-microbial B-defensin 2, B-defensin 3, S100 proteins, and lipocalin. IL-17 functions as a potent pro-inflammatory cytokine that initiates keratinocytes to release neutrophil-attracting CXC chemokines like CXCL5, CXCL1, and CXCL8/IL8 along with CCL20 that draw CCL6+ cells into areas of inflammation. IL23 and Th17 cells are found to be markedly abundant in psoriasis lesions. It explains the production of mainly psoriasin and other innate defense molecules, which are typical of psoriasis.

IL12/23 Pathway

IL12 and IL23 have been known to be as main components in the pathogenesis of psoriasis as these cytokines are released by various immune cells and promote T cell response by directing the differentiation of naïve T cells into TH17, and TH1 cells, respectively.

The common subunit P40 of IL23 and IL12 is over-expressed in psoriatic plaques. IL12 affects the development of TH1 cells that release TNF-alpha, IFN-Y, and IL2, while IL23 drives the production of Th17 cells that produce TNF-alpha, IL17, and IL22. IL23 has more influence over the induction of autoimmune-mediated inflammation than the IL12/TH1 axis; thus IL23/Th17 axis is the predominant pathway involved in psoriasis.

Innate Immunity and the Role of Keratinocytes

DCs (myeloid and plasmacytoid), NK cells, neutrophils, innate lymphoid cells, and epidermal keratinocytes are a few cells involved in innate immunity [14]. Keratinocytes express antimicrobial proteins, e.g. B defensins which have chemotactic properties. Also, these keratinocytes secrete signaling molecules, e.g. IL-6, IL8, IL-1, and TNF-alpha, and are a source of growth factors for angiogenesis.

Aryl Hydrocarbon Receptor (AhR)

AhR is the cytosolic ligand-activated receptor and transcription factor that is mainly expressed in skin cells, and AhR in cutaneous vascular endothelial cells (VECs) is crucial for the emergence of psoriasis [15].

Treatment

Psoriasis treatment options have been evolving over a period of time, and the condition was once primarily thought of as a cosmetic skin condition. The traditional treatment options include coal tar, anthralin, arsenic, and salicylic acid. Methotrexate, retinoids, vitamin D analogs, and cyclosporine have also played their role in the management of psoriasis.

New biologics have developed dramatically over the past 10 years, and by blocking particular cytokines, they provide more effective and disease-specific treatment. The treatment includes both topical and systemic therapies.

Topical therapy

Corticosteroids

Form the cornerstone of topical regimen for psoriatic patients. Very potent and potent topical corticosteroids are known as the first-line topical agent based on their cost-effectiveness. For the plaque psoriasis occurring in areas of thick skin, e.g. palms and soles, potent and very potent corticosteroids are suitable; however, in areas such as the face, flexures, and neck, mild to moderate potency steroids are used. Anti-inflammatory, vasoconstriction, anti-proliferative, and immunosuppression are a few mechanisms of the actions of steroids. Topical corticosteroids are available in various formulations, of which ointment has the highest efficacy.

Although not common, long-term use can be compounded by potential adverse problems such as tachyphylaxis, hypothalamic-pituitary-adrenal axis suppression, and local cutaneous side effects like atrophy and telangiectasia [16].

Vitamin D3 Analogs

Due to their therapeutic efficacy and limited adverse effects, vitamin D3 analogs are considered as first-line topical therapy for plaque psoriasis, scalp psoriasis, and psoriasis occurring in intertriginous areas. Reduction of the proliferation of keratinocytes, modulation of epidermal differentiation, and shifting of cytokine profile from Th1 towards Th2 are plausible mechanisms of action. Analogs of vitamin D3 are frequently used in monotherapy or in combination therapy. The two synthetic analogs include calcipotriol and tacalcitol. Burning sensation, irritation, and hypercalcemia are a few side effects related to excessive usage. Vitamin D3 analog should always be applied following phototherapy since UV radiation can cause degradation of vitamin D3 analog.

Topical Retinoids

Topical tazarotene gel of 0.1% and 0.05% is FDA-approved for the mild case to moderate plaque psoriasis. Topical retinoids normalize keratinocyte differentiation and proliferation. It is considered as a regimen for second-line option either as combination therapy or monotherapy for mild-moderate plaque psoriasis.

Topical Calcineurin Inhibitors

Tacrolimus, 0.1% ointment, has been found to have a defined role in facial, genital, and inverse psoriasis.

Combined therapy

A Cochrane analysis has revealed that the combination of betamethasone dipropionate and calcipotriol is more useful for treating psoriasis than any one of the treatments alone [17]. Also, clinical studies have also shown a decreased incidence of negative outcomes when topical corticosteroids and vitamin D3 mimics are used concurrently or sequentially [18].

Systemic therapy

Indications to start systemic therapy includes the dermatology life quality index (DLQI) and psoriasis area and severity index (PASI). Body surface area associated with psoriatic arthritis, significant cosmetic or psychosocial impact, involvement of high-impact sites, e.g. palms, soles, nails, and genitalia.

Phototherapy

A common regimen for moderate to severe psoriasis is phototherapy, mainly when the condition is refractory to topical medications [16]. The various forms of phototherapy include psoralen plus UVA (PUVA) and narrow-spectrum UVB (NB-UVB). Due to its safety benefits and efficacy, which have been demonstrated in numerous RCTs, NB-UVB therapy is frequently utilized as the first-line regimen as monotherapy or in combination for moderate cases to severe psoriasis [19]. NB-UVB can be used in all variants of psoriasis except pustular and erythrodermic. The thin plaques of guttate psoriasis and sebopsoriasis respond most readily to UVB. NB-UVB therapy can be given to patients of any age group, including children and pregnant women. There is no evidence that NB-UVB increases the risk of skin malignancy [20]. PUVA therapy can be used for all types of psoriasis. T-cell apoptosis and inhibition of keratinocyte hyperproliferation are the two major mechanisms of action. The dose of 8 methoxy psoralen is 0.6-0.8mg/kg orally, to be taken 1-3 hours before exposure to UVA. The frequency of PUVA therapy is 2-3 times a week. According to the recommendations, patients should not receive more than 200 treatment sessions of PUVA in a lifetime. Nausea, vomiting, PUVA itch, and increased risk of non-melanoma skin cancers are a few side effects of PUVA.

Acitretin

Acitretin is the only retinoid FDA-approved for the treatment of psoriasis. It causes inhibition of keratinocyte proliferation, inhibits the Th17 cells, and increases the activity of T regulatory cells. Since it is not an immunosuppressant, it can be used in conditions where systemic immunosuppressants are contraindicated, e.g. malignancy. Systemic retinoids are used as a monotherapy for erythrodermic psoriasis, pustular psoriasis, and severe psoriasis that cannot be managed by topical or photochemotherapy. Mucocutaneous dryness, hyperlipidemia, and transaminitis are a few typical adverse effects. It is advised that women who are of reproductive age or who may get pregnant avoid taking the drug for three years after stopping the medicine [19].

Methotrexate

This is a folate antagonist and an inhibitor of the enzyme dihydrofolate reductase. It is the first-line systemic drug used in cases of plaque psoriasis having more than 20% of body surface area, especially if there is co-existing psoriatic arthritis [16].

Hepatotoxicity is a well-known adverse consequence. Nausea, vomiting, and mucositis are a few other important side effects.

Cyclosporine

Calcineurin inhibitor, cyclosporine, is used to treat sudden severe flare of psoriasis, erythrodermic and pustular psoriasis, and moderate-severe psoriasis or disabling psoriasis who have failed other systemic therapy [16].

Rapid mechanism of action and reduced potential for myelosuppression and hepatotoxicity are a few advantages over other systemic agents. Nephrotoxicity, diastolic hypertension, increased triglyceride levels, gingival hyperplasia, hypomagnesemia, and hyperkalemia are a few of the side effects [19].

Biologic Therapy

When regular systemic medications do not produce a supportive response, they are not tolerated due to side effects or are inappropriate because of comorbidities; biologics come to play as the therapeutic option in psoriasis [21]. The selection of therapy is based on clinical requirements, advantages and disadvantages, patient preferences and cost-effectiveness.

Biologic therapies available currently for psoriasis

TNFα Inhibitors

It includes etanercept, infliximab, adalimumab, certolizumab, and golimumab. Certolizumab pegol is a TNF inhibitor that is PEGylated and Fc-free. It demonstrates limited placental transfer from mothers to neonates because it does not combine the neonatal Fc receptor for IgG (FcRn) [22]. Reactivation of latent tuberculosis, hepatitis C and B, drug-induced lupus and demyelinating central nervous system illnesses, and paradoxical reactions such as psoriasis are a few serious adverse effects observed [23].

IL23 Inhibitors

Guselkumab, tildrakizumab, and risankizumab inhibit the P19 subunit of IL23, while ustekinumab targets the common P40 subunit of IL12 and IL23. Nasopharyngitis, upper respiratory tract infection, and major adverse cardiovascular events (MACE) are the predominant side effects [24].

IL17 Inhibitors

Ixekizumab and secukinumab is the inhibitor of IL17, while brodalumab is an IL17 receptor antagonist. The important side effects include nasopharyngitis, neutropenia, candidiasis, inflammatory bowel disease, and an increased risk of suicide [24].

RORγt Inhibitors

The orphan nuclear receptor gamma t (RORt or RORc2) is an important transcription factor for the progression of Th17 cells [25]. A thorough approach to treating psoriasis involves preventing RORt activation [25].

IL36 Receptor Antagonist

In psoriasis, IL36, a member of the IL1 superfamily, is crucial for attracting and stimulating neutrophils and Th17 cells [26]. Spesolimab is humanized, novel, selective antibody that prevents the activation of the interleukin-36 receptor (IL-36R), it is being studied for the regimen of generalized pustular psoriasis flares.

Sphingosine-1-Phosphate (S1P) Agonist

Ceramidase converts ceramide into sphingosine, and sphingosine kinase converts sphingosine into S1P. S1P is the bioactive lipid metabolite that shows its actions by engaging 5 G-protein-coupled receptors (S1PR1-S1PR5). S1P receptors are used in many physiological, cellular events involving lymphocyte/hematopoietic cell trafficking [27,28]. A novel orally active S1P 1 modulator desensitizes the peripheral pathogenic lymphocytes to egress signal from secondary lymphoid organs and mainly the thymus, thus presenting a promising drug for psoriasis treatment.

Rho-Associated Kinase (ROCK2) Inhibitor

Rho family kinases have both ROCK2 and ROCK1, are serine-threonine kinases initiated by Rho GTPases, and mediate the process of phosphorylation of downstream targets in cells [29]. Targeted inhibition of Rho-associated kinase (ROCK)2 down-regulates the pro-inflammatory T cell response while it increases the regulatory arm of the immune response autoimmunity models in animals and Th17-skewing human cell culture in-vitro [30].

The AhR Agonist

Tapinarof naturally occurring polyphenols produced by entomopathogenic nematode symbionts and an AhR agonist [31].

Janus Kinase (JAK) Inhibitors

Janus kinases inhibitors are molecules that target activators of transcription (JAK/STAT) and Janus kinases-signal transducers. They prohibit this intracellular signal way and block the proinflammatory cytokines gene transcription that has a main role in the pathogenesis of psoriasis [32].

## Conclusions

In spite of its increased occurrence and important impact on quality of life, psoriasis is a multisystem inflammatory disease that is both underdiagnosed and underrated. Along with affecting the skin and joints, psoriasis is also linked to a wide range of significant medical and psychological comorbidities that necessitate prompt treatment to improve long-term outcomes. In this study, we have emphasized on few important aspects of pathogenesis e.g. antimicrobial peptides, the implication of DCs, the IL23/IL17 axis, along with the role of innate immunity and AhR. Additionally, the article shows the cornerstones in the management of psoriasis along with the various upcoming therapies in the pipeline and those under therapeutic trials enumerating a few such as ROCK inhibitors, JAK inhibitors, AhR agonists, and S1P agonists.

## References

[1] Pariser DM, Bagel J, Gelfand JM (2007). National Psoriasis Foundation clinical consensus on disease severity. Arch Dermatol.

[2] Lal S (1966). Clinical pattern of psoriasis in Punjab. Indian J Dermatol Venereol.

[3] Mehta TK, Shah RN, Marquis L (1976). A study of 300 cases of psoriasis. Indian J Dermatol Venereol Leprol.

[4] Hu P, Wang M, Gao H, Zheng A, Li J, Mu D, Tong J (2021). The role of helper T cells in psoriasis. Front Immunol.

[5] AlShobaili HA, Shahzad M, Al-Marshood A, Khalil A, Settin A, Barrimah I (2010). Genetic background of psoriasis. Int J Health Sci (Qassim).

[6] Fan X, Yang S, Huang W (2008). Fine mapping of the psoriasis susceptibility locus PSORS1 supports HLA-C as the susceptibility gene in the Han Chinese population. PLoS Genet.

[7] Balak DM, Hajdarbegovic E (2017). Drug-induced psoriasis: clinical perspectives. Psoriasis (Auckl).

[8] Baker BS, Brown DW, Fischetti VA, Ovigne JM, Porter W, Powles A, Fry L (2001). Skin T cell proliferative response to M protein and other cell wall and membrane proteins of group A streptococci in chronic plaque psoriasis. Clin Exp Immunol.

[9] Farber EM, Nickoloff BJ, Recht B, Fraki JE (1986). Stress, symmetry, and psoriasis: possible role of neuropeptides. J Am Acad Dermatol.

[10] Michaëlsson G, Gustafsson K, Hagforsen E (2006). The psoriasis variant palmoplantar pustulosis can be improved after cessation of smoking. J Am Acad Dermatol.

[11] Mitra A, Raychaudhuri SK, Raychaudhuri SP (2012). Functional role of IL-22 in psoriatic arthritis. Arthritis Res Ther.

[12] Zdanowska N, Kasprowicz-Furmańczyk M, Placek W, Owczarczyk-Saczonek A (2021). The role of chemokines in psoriasis - an overview. Medicina (Kaunas).

[13] Victor FC, Gottlieb AB (2002). TNF-alpha and apoptosis: implications for the pathogenesis and treatment of psoriasis. J Drugs Dermatol.

[14] Sweeney CM, Tobin AM, Kirby B (2011). Innate immunity in the pathogenesis of psoriasis. Arch Dermatol Res.

[15] Zhu Z, Chen J, Lin Y (2020). Aryl hydrocarbon receptor in cutaneous vascular endothelial cells restricts psoriasis development by negatively regulating neutrophil recruitment. J Invest Dermatol.

[16] Papp K, Gulliver W, Lynde C, Poulin Y, Ashkenas J (2011). Canadian guidelines for the management of plaque psoriasis: overview. J Cutan Med Surg.

[17] Mason AR, Mason J, Cork M, Hancock H, Dooley G (2013). Topical treatments for chronic plaque psoriasis: an abridged Cochrane systematic review. J Am Acad Dermatol.

[18] Scott LJ, Dunn CJ, Goa KL (2001). Calcipotriol ointment. A review of its use in the management of psoriasis. Am J Clin Dermatol.

[19] Menter A, Korman NJ, Elmets CA (2009). Guidelines of care for the management of psoriasis and psoriatic arthritis. Section 3. Guidelines of care for the management and treatment of psoriasis with topical therapies. J Am Acad Dermatol.

[20] Weischer M, Blum A, Eberhard F, Röcken M, Berneburg M (2004). No evidence for increased skin cancer risk in psoriasis patients treated with broadband or narrowband UVB phototherapy: a first retrospective study. Acta Derm Venereol.

[21] Menter A, Gottlieb A, Feldman SR (2008). Guidelines of care for the management of psoriasis and psoriatic arthritis: Section 1. Overview of psoriasis and guidelines of care for the treatment of psoriasis with biologics. J Am Acad Dermatol.

[22] Mariette X, Förger F, Abraham B (2018). Lack of placental transfer of certolizumab pegol during pregnancy: results from CRIB, a prospective, postmarketing, pharmacokinetic study. Ann Rheum Dis.

[23] Gall JS, Kalb RE (2008). Infliximab for the treatment of plaque psoriasis. Biologics.

[24] Tokuyama M, Mabuchi T (2020). New treatment addressing the pathogenesis of psoriasis. Int J Mol Sci.

[25] Tang L, Yang X, Liang Y, Xie H, Dai Z, Zheng G (2018). Transcription factor retinoid-related orphan receptor γt: a promising target for the treatment of psoriasis. Front Immunol.

[26] Ganesan R, Raymond EL, Mennerich D (2017). Generation and functional characterization of anti-human and anti-mouse IL-36R antagonist monoclonal antibodies. MAbs.

[27] Kunkel GT, Maceyka M, Milstien S, Spiegel S (2013). Targeting the sphingosine-1-phosphate axis in cancer, inflammation and beyond. Nat Rev Drug Discov.

[28] Park SJ, Im DS (2017). Sphingosine 1-phosphate receptor modulators and drug discovery. Biomol Ther (Seoul).

[29] Riento K, Ridley AJ (2003). Rocks: multifunctional kinases in cell behaviour. Nat Rev Mol Cell Biol.

[30] Zanin-Zhorov A, Weiss JM, Trzeciak A (2017). Cutting edge: selective oral ROCK2 inhibitor reduces clinical scores in patients with psoriasis vulgaris and normalizes skin pathology via concurrent regulation of IL-17 and IL-10. J Immunol.

[31] Campione E, Cosio T, Di Prete M, Lanna C, Dattola A, Bianchi L (2021). Experimental pharmacological management of psoriasis. J Exp Pharmacol.

[32] Słuczanowska-Głąbowska S, Ziegler-Krawczyk A, Szumilas K, Pawlik A (2021). Role of Janus kinase inhibitors in therapy of psoriasis. J Clin Med.

